# Present and Future of the Use of Artificial Intelligence in Orthodontics

**DOI:** 10.3390/bioengineering13030263

**Published:** 2026-02-25

**Authors:** Ilaria Tucci, Elena Gimondo, Elena Jovanova, Angela Angjelova, Gaetano Isola, Cristina Grippaudo

**Affiliations:** 1 Dipartimento Universitario Testa Collo ed Organi di Senso, Università Cattolica del Sacro Cuore, 00168 Rome, Italy; ilaria.tucci01@gmail.com (I.T.); elena.gimondo14@gmail.com (E.G.); 2 Department of General Surgery and Surgical-Medical Specialties, Unit of Periodontology, School of Dentistry, University of Catania, 95123 Catania, Italyangela.angjelova@students.stomfak.ukim.mk (A.A.); gaetano.isola@unict.it (G.I.); 3 University Dental Clinical Center St. Pantelejmon, Faculty of Dentistry, Ss. Cyril and Methodius University in Skopje, 1000 Skopje, North Macedonia; 4 International Research Center on Periodontal and Systemic Health “PerioHealth”, University of Catania, 95123 Catania, Italy; 5 UOC di Clinica Odontoiatrica, Dipartimento di Neuroscienze, Organi di Senso e Torace, Fondazione Policlinico Universitario A. Gemelli, IRCCS, 00168 Rome, Italy

**Keywords:** artificial intelligence, orthodontics, diagnosis, therapy, compliance

## Abstract

Artificial intelligence (AI) has penetrated many aspects of orthodontic clinical practice over the past decade. This narrative review aims to highlight all the areas where AI has already entered clinical routine and how it can further improve orthodontic practice in the future. AI is present in diagnostic procedures, facilitating record keeping and supporting the study of patient radiographic and photographic image characteristics. It also underpins the creation and analysis of digital impressions. AI has also become part of patient communication processes, helping to share treatment decisions and facilitating treatment monitoring. Technology and the availability of clinical data also enable the use of AI in diagnostic processes. Currently, clinicians must evaluate AI-provided clinical suggestions before accepting them, as is the case with clear aligner treatments, for example. Research on AI in dentistry, reported in this article, promises to increase the precision of diagnostic measurements and facilitate clinicians’ treatment decisions.

## 1. Introduction

The growing interest in technologies using artificial intelligence in orthodontics stems from clinicians’ desire to achieve treatment goals more quickly and safely. Technology is being sought to improve the many aspects of orthodontic diagnosis and treatment that can be influenced by human error. To better understand this concept, it is helpful to recall how an orthodontist’s training process works. The biological basis of orthodontics lies in the general aspects that regulate craniofacial growth and tooth movement. This knowledge is complemented by information on therapeutic methods and their mechanisms of action. The training process continues with the acquisition of skills in the use of diagnostic tools and the correct interpretation of data. For these aspects, reference is made to the classification systems for malocclusions. Finally, the training program is developed, and the general principles of diagnosis and treatment must be applied to the specific clinical case being examined. For this reason, experience is a fundamental ingredient in an orthodontist’s training, and it is not limited to clinical practice but must also encompass the relationship with the patient and their expectations.

Artificial intelligence, which simulates the human learning process and leads to fast data processing, raises great expectations and has become a hot topic in leading scientific journals and many orthodontic conferences around the world.

In the early 2000s, the use of artificial intelligence in medicine began to be proposed as a tool to support and accelerate progress in diagnostics and clinical practice [1]. In the years that followed, integrated research in the medical and computer sciences produced numerous tools that use AI. This also applies to the dental field, as a support for several clinical aspects, such as diagnosis, clinical decisions, treatment plans, and prognosis prediction. As indicated by Gandedkar et al. [2], in the decade 2009–2019, the field of orthodontics in particular became increasingly digital: there was a notable advancement in 3D technologies, computer-aided design (CAD), and computer-aided manufacturing (CAM).

A systematic review of AI across dental specialties confirmed that it helps reduce chair-time, improve infection control, and aids in diagnosis and clinical decision-making, predicting dental failures [3]. The medical field in which AI has shown the best results is diagnostic imaging. It is demonstrated that artificial intelligence has utility in diagnosing lesions, localizing landmarks, and segmenting jawbone, airway, and sinus structures from CBCT scans with satisfactory accuracy for clinical use. To this end, there is a progressive improvement in algorithms for the diagnostics of dental images [4].

Developing algorithms that propose therapeutic solutions is only possible with adequate clinical datasets [5]. For example, there are proposals for tools to assess craniofacial growth, which can be trained using radiographic images readily available in orthodontic archives. Another approach involves the clinical decisions underlying orthodontic extractions, based on cephalometric data [6]. However, the most widely used application is to program orthodontic movements with clear aligners, because manufacturers have a vast number of clinical cases at their disposal [7].

If AI is used by orthodontists, it is being consulted even more by ordinary people. The era we live in, characterized by widespread internet use, has favored the search for medical answers online. Recently, many have been looking for answers to their questions from chatbots because the responses are more comprehensive and interactive. These activities should be considered as elements of the orthodontist–patient relationship, because thanks to ease of access, they will become increasingly widespread.

**Figure 1:**
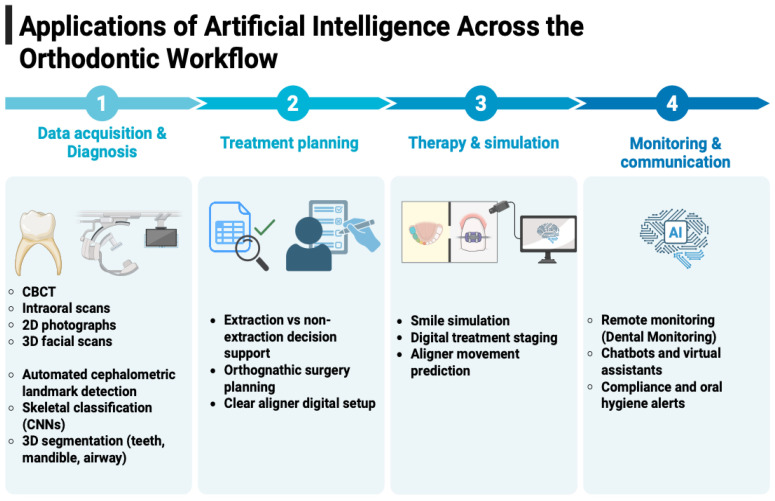
Overview of artificial intelligence applications across the orthodontic workflow, from data acquisition and diagnosis to treatment planning, therapy simulation, patient monitoring, and communication. Created with https://BioRender.com.

In this work, we propose a review aimed at specifically identifying the real usefulness and true involvement of AI in various areas of orthodontics, including diagnosis, therapy, and patient monitoring. Finally, we identify potential limitations and areas for improvement to maximize utility and support clinical practice, without completely replacing the professional role of the dentist. The search for articles cited in this narrative review was conducted using Orthodontics and Artificial Intelligence as keywords and narrowing the selection to the years 2019 to January 2026, to provide a current overview of the state of the art in this field. To contextualize the subsequent sections, Figure 1 illustrates the key areas where AI supports orthodontic diagnosis, treatment planning, and patient management. Table 1 summarizes the current clinical readiness of AI applications in orthodontics, highlighting which tools are in research, pilot, or routine use.

## 2. Use of AI for Radiographic Analysis

The cephalometric study of the patient, an essential element for orthodontic diagnosis, is an operation that requires time and experience. The technological advances applied to cephalometric analysis have led most orthodontists to use cephalometric analysis software. These tools, which speed up calculations and increase measurement accuracy, do not use AI and speed up the cephalometric analysis. AI-based systems, thanks to computer vision technologies, allow for the identification of structures and selection of landmarks, without any intervention by the orthodontist [8].

A standard cephalometric analysis requires the positioning of anatomical landmarks, an initial phase that, if performed manually by the operator, already requires time and concentration to avoid errors (Figure 1). In AI-assisted programs, the machine acquires the experience necessary to identify anatomical structures and landmarks. The question addressed in numerous studies on the topic concerns the reliability of landmark identification with automatic systems. The most commonly used method for quality assurance is to compare the results with cephalometric tracings performed manually by expert operators.

Below, we present the most significant studies on the topic, which highlight the quality achieved by new methods and their potential for improvement.

Kunz et al.’s research highlights above all the advantages of using new cephalometric analysis systems [9]. From the comparison between their artificial intelligence algorithm specialized in automated cephalometric analysis and the analysis conducted by human experts, no significant differences emerged, demonstrating that the algorithm produced by them has a quality comparable to the experience of specialist examiners.

Some machine learning algorithms were created with the human brain as inspiration, in particular, the biological neural network [10,11]. For image analysis, the convolutional neural network (CNN) technique has gained ground [12]: to avoid inter- and intra-variability errors as much as possible and to reduce the execution time of cephalometric traces, Yu et al. [13] applied a multimodal CNN directly to lateral cephalograms for skeletal class diagnosis, bypassing landmark tracing altogether. Their model reached 96.40% vertical-classification accuracy, confirming that end-to-end AI can match or exceed expert performance in diagnostic tasks.

A few years later, a different AI paradigm was proposed by Jiang et al. [14]: a two-stage convolutional neural network—named “CephNet”—trained on 9870 multi-institutional cephalograms. It achieved an average landmark error of 0.94 ± 0.74 mm and 89.33% classification accuracy for derived measurements. The markedly lower error reflects the power of deep learning when supplied with large, heterogeneous datasets.

AI also showed accuracy in a further study [15] that compared cephalometric landmark placement with deep learning (DL) with manual placement by experts. In conclusion, the deep learning algorithms showed an acceptable level of accuracy, but it remains unclear whether the algorithms are similar, superior, or inferior to the clinicians themselves, due to the heterogeneity in placing different landmarks.

However, there is also a possible negative aspect to consider that can compromise the accuracy of the cephalometric analysis performed by artificial intelligence software. The study by Polizzi et al. [16] aimed to compare cephalometric measurements performed with automated and non-automated software in cases of correct and incorrect positioning of the head. The result showed a good level of consistency for angular measurements, while linear measurements were more susceptible to errors. Position errors drastically reduced the accuracy of the measurements; in particular, the least concordant value between the software programs was Go-Me. This article highlights one of the disadvantages of using AI in cephalometric analysis: in case of head positioning errors, the software proceeds to take measurements that will be inaccurate.

The review by Hendrickx et al. [17] aimed to investigate the efficiency and accuracy of automatic identification of cephalometric landmarks performed by artificial intelligence systems in both 2D on lateral cephalometric radiographs and 3D on CBCT. Out of a total of 34 publications (27 on 2D images and seven on 3D ones), it was found that, on 2D images, the error was below the clinically acceptable threshold of 2 mm. The meta-analysis could not be carried out for 3D images due to significant heterogeneity among studies. However, the qualitative synthesis showed an average error in landmark detection on 3D images ranging from 1.0 to 5.8 mm. In conclusion, automatic detection of cephalometric landmarks using AI has shown good potential, being accurate and faster, but these systems could still benefit from further improvements.

The umbrella review by Polizzi et al. [18] also aimed to analyze the performance of AI in the automatic identification of cephalometric landmarks. An analysis of the eleven systematic reviews included in the study revealed that expert orthodontist supervision is still required to identify cephalometric points: in fact, despite improved performance, artificial intelligence has gone beyond the recommended error range for most cephalometric landmarks, and in the future, this automatic analysis software should be improved.

Artificial intelligence has also been applied to the analysis of orthodontically useful 3D radiographs, such as CBCT. Some authors have proposed models for the automatic localization of cephalometric landmarks in 3D space, and not two-dimensionally on cephalometry. This is the case of Montufar et al. [19], who introduced an active-shape-model (ASM) that locates cephalometric landmarks in 3D space: their analysis of 24 head CBCTs revealed a mean localization error of 3.64 mm for 18 anatomical points with this method, with the largest discrepancies around the Porion and Sella regions, where image definition was lower. This work proved that a fast 2D search can replace exhaustive volume analysis, yet the residual error limits direct clinical adoption.

A later hybrid strategy built on the same ASM concept, but added a 3D knowledge-based algorithm on cropped sub-volumes for landmark local adjustment. The same sample size produced a mean location error of 2.51 mm, an improvement that the authors attributed to the local adjustment stage [20]. The authors concluded that their method of automatically localizing landmarks on CBCTs is useful for 3D cephalometric analyses and a step forward compared to conventional 2D cephalometry.

Three-dimensional imaging is useful, especially for placing soft tissue landmarks, as in two-dimensional analysis, the emphasis is often placed on hard tissues, which are better reproducible than the soft ones [21].

In the field of three-dimensional radiography, artificial intelligence software can identify anatomical structures such as teeth and bones in three-dimensional radiographic images for diagnostic and therapeutic study purposes. Nowadays, artificial intelligence can also be used for so-called segmentation, the technique that allows us to distinguish a specific element by excluding the remaining anatomical structures in three-dimensional models: for example, it is possible to separate the teeth from the surrounding bones, calculate the volume of the airways, and precisely study the position of the dental elements.

An automatic 3D jaw segmentation model has been presented by Spampinato et al. [22] for the identification of facial asymmetries. The results were very similar to those obtained by the experts, but they gained a lot of time with the method they proposed.

The more recent study by Lo Giudice et al. [23] demonstrates that a deep learning convolutional neural network (CNN) can segment the mandible from CBCT data with expert-level precision but at a much faster speed. Both studies illustrate how the labor-intensive manual process of mandibular segmentation can become rapid and reproducible with the use of automatic or AI-based methods.

Regarding tooth segmentation in CBCT images, AI methods have shown excellent results, but there are still some problems, such as the too small size of the training data and the non-uniformity of the evaluation metrics, that still need to be improved [24]. Overall, these studies underscore the increasing role of AI in radiographic analysis, particularly in automating segmentation and enhancing efficiency and reproducibility. Building on progress in hard-tissue imaging, recent work has shifted toward soft-tissue evaluation using 3D facial imaging.

## 3. Use of AI for Stereophotogrammetric Images Analysis

In the medical field, the improvement of three-dimensional imaging technologies is highly appreciated, especially for aesthetic evaluations in the orthodontic field, but also in maxillofacial surgery. The positioning of the landmarks can, in fact, be done on the soft tissue using stereophotogrammetric images, and it is possible to precisely measure facial volumes and areas. Additionally, it may be helpful to create a virtual patient by matching the face in a 3D image with a CBCT and a scanned model of the teeth. The study by Baysal et al. [25] analyzed intra- and inter-examiner repeatability and reproducibility in the placement of soft tissue landmarks on stereophotogrammetric images: apart from a single point (the labial superior one—which had a difference of less than 0.5 mm intra-examiner), all the landmarks showed moderate reproducibility, but with differences of less than 1 mm, showing acceptable reproducibility.

Artificial intelligence, especially deep learning and convolutional neural networks (CNNs), is rapidly reshaping the analysis of stereophotogrammetric (3D) facial images in orthodontics.

A study of 408 3D adult face images analyzed the capability of a framework for automatically locating landmarks on three-dimensional images, specifically a patch-based CNN method [26]: 37 landmarks were taken from 3D images of faces by an expert, and then the same sets of landmarks were automatically identified. The system achieved an overall mean localization error of 0.83 ± 0.49 mm, comparable to the intra-operator error of 0.56 ± 0.69 mm, showing the effectiveness of this method in detaining skin landmarks.

A DiffusionNet workflow on 2897 3D facial photographs about 10 cephalometric facial landmarks localization (exocanthions, endocanthions, nasion, nose tip, alares, and cheilions) yielded a mean precision of 1.69 ± 1.15 mm, comparable to the inter-observer variability of manual annotation (1.31 ± 0.91 mm) [27]. Due to its high accuracy, the automated method developed in this study has the potential to be used on larger data sets for the analysis of facial soft tissues to assess facial deformities, development, and asymmetries for orthodontic diagnostic purposes.

An automatic landmark process was proposed by the study by Baski et al. [28] on 3D images of a sample of 30 subjects between 9 and 17 years of age. The automated algorithm consistently identified midsagittal landmarks (e.g., pronasale, subnasale, subspinale, labiale superius, stomion) with ≤2 mm error, whereas peripheral points (e.g., exocanthion, glabella) showed larger discrepancies.

The systematic review by Al-Baker et al. [29] analyzed 14 studies regarding the automatic 3D localization of facial landmarks, concluding that deep learning models are the ones that provide the best performance. However, according to this review, automated 3D facial image landmarking models are not clinically comparable to manual ones, and in the future, we should focus on implementing these systems while considering that the quantity and quality of the data influence the analysis results.

In conclusion, artificial intelligence can also be used for orthodontic purposes for facial aesthetic assessments on 3D images and stereophotogrammetry, facilitating the visualization of a complete and correct diagnosis in a short time and allowing for the best possible preparation of a correct treatment plan. Building on AI applications in facial analysis, recent research has also focused on its role in predicting craniofacial growth, a key component of long-term orthodontic planning.

## 4. Use of AI for Craniofacial Growth Prediction

Prediction of craniofacial growth is useful at all stages of orthodontic treatment: it helps to understand the causes of malocclusion, the possibilities and limitations of therapies, and the long-term prognosis. Advances in this field of research are correlated with technological developments. The first methods for predicting facial growth relied on the superimposition of cephalometric tracings (Bolton standard, Ricketts). Björk used metal implants to measure maxillary growth on cephalometries [30]. His research led to the definition of anterior and posterior growth. Research by Petrovic et al. [31], combining biological data from condylar cartilage cell cultures with cephalometric data, led to the identification of 33 rotational types of facial growth and six auxological categories regarding the developmental potential of the upper and lower jaws. This knowledge, however, cannot establish facial growth trends with certainty, but only provides diagnostic guidance.

Nowadays, the possibility of training machines by providing adequate models has generated the hypothesis that, automatically, it is possible to obtain information on the facial growth of the patient under examination.

Zhang et al. [32] developed a CNN model to predict the progression of mandibular growth in children with anterior crossbite, categorizing it as normal or underdeveloped. The deep learning CNNs applied to pre-treatment cephalograms classified mandibular development with 85% accuracy and outperformed junior clinicians in sensitivity and specificity. According to this study, the deep learning CNN model could predict the growth trend of the mandible in children’s anterior crossbite with relatively high accuracy using cephalometric images.

A further study used a CNN model to investigate growth prediction by analyzing the lateral cephalometries of 198 Japanese preadolescents in Class between 8 and 10 years [33]. The model achieved acceptable mean prediction accuracy errors for most hard-tissue landmarks, but performed relatively lower for soft-tissue landmarks.

Other studies have used automatic machine learning (ML) models, such as that of Myers et al. [34], who analyzed the cephalometries at pre- and post-pubertal stages. This study aimed to predict long-term growth-related changes in the skeleton and dental relationships within the craniofacial complex, specifically using Lasso, Random Forest, and Support Vector Regression (SVR).

ML models showed higher accuracy for skeletal relationships than for dental relationships over the 8-year growth period. The most important predictors of post-pubertal values were found to be the pre-pubertal values of measurements and sex.

One of the machine learning models in the literature aimed to predict post-pubertal mandibular length and *Y*-axis in 176 Class I females: ML methods were identified as being able to predict mandibular length within 3 mm and the *Y*-axis within 1 degree. All ML algorithms were shown to be equally accurate, with the exception of the multilayer perceptron regressor [34,35].

Kim et al., in their analysis, aimed to identify the best model to predict craniofacial growth on the cephalometries of 59 Japanese children never treated orthodontically, concluding that among all the machine learning models, LASSO added the highest accuracy [35].

Several ML methods were also studied in the analysis by Zakhar et al. [36], but this time in Class II males. The tested ML algorithms accurately predicted post-pubertal mandibular length within a range of 2.5 mm and the *y*-axis within 1°.

In the analysis of cephalometric data of 163 Class I males with different ML algorithms, all of them showed an accuracy range from 95.8% to 97.64% for post-pubertal mandibular length prediction and from 96.60% to 98.34% for *Y*-axis growth prediction [37].

The ML attempt taken by Kazmierczak et al. [38] to predict facial growth direction achieved 71–75% classification accuracy, limited by a small sample size, different sources of data, class imbalance, and a reliance on 2D landmarks.

As stated by Kwon et al. [39], developing AI models to predict craniofacial growth, including growth data from a considerable number of subjects, could improve the prediction method. However, craniofacial growth prediction models, even when performed on a large amount of data, remain a significant challenge.

AI has sometimes shown lower accuracy in predicting growth in soft tissues compared to hard tissues [32] and also in predicting dental inclinations and lower-face height [33].

Despite all the positive aspects mentioned, the craniofacial growth prediction performed by AI also has some limitations. It would certainly be useful to increase the total size of the analyzed dataset, but also to include additional clinical and family characteristics in the patients’ medical history [31]. All the research described here has not yet produced systems that can be routinely used in clinical practice. The topic of craniofacial growth prediction remains unresolved, and orthodontists lack scientific evidence demonstrating the impact of orthodontic treatment on growth. The critical point with all systems is that the data provided to the machines to train them to understand facial growth is based only on the appearance of clinical cases, and not on biological characteristics, as Petrovic, for example, attempted. This leads to indications that are based solely on the number of training samples used but may not be useful for predicting the growth of the patient being treated.

## 5. Use of AI for Treatment Purposes

Artificial intelligence is increasingly applied in clinical medicine to enhance diagnostic accuracy and predict treatment outcomes through the analysis of large-scale data from imaging, pathology, genomics, and medical records (Figure 1). In orthodontics, AI supports several key tasks, including automated anatomical landmark detection, cephalometric analysis, skeletal classification, extraction decision-making, orthognathic surgery planning, and forecasting treatment results. These applications have been shown to reduce operator variability, minimize human error, and improve workflow efficiency compared to traditional manual methods [39,40].

Among the most documented applications is extraction decision-making. The topic of extractions in orthodontics is controversial from many perspectives. First, some orthodontic techniques are considered more or less extractive, based on the presumed possibility of modifying the alveolar bone. Nonetheless, all orthodontists agree that in some cases, extractions are fully justified. Many factors influence the decision on which teeth to extract, as described by Travess et al. [40].

Researchers have attempted to train artificial intelligence systems to reason according to some of these dictates. Seok-Ki Jung and Tae-Woo Kim’s research is in this vein and demonstrated that neural network models can replicate expert reasoning in nonsurgical extraction cases, integrating variables such as extraction position, crowding, and protrusion to improve predictive accuracy [41].

Similarly, the study by Peilin Li et al. [42] showed that such models achieve high accuracy (up to 94%) and area under the curve (AUC) values (up to 0.98), with key predictive features including dental crowding, sagittal skeletal relationships (e.g., ANB angle), incisor inclination, and facial profile. Artificial intelligence, more specifically a multilayer perceptron neural network, can be used to automatically generate orthodontic treatment plans by first deciding whether extraction is necessary and then predicting the specific extraction model or anchoring strategy to use. The model attained 94% accuracy for the extraction–nonextraction treatment prediction (with an AUC of 0.982, sensitivity 94.6%, specificity 93.8%) and reached 84.2% and 92.8% accuracy for extraction-pattern and anchorage-pattern prediction, respectively. The most important predictors identified by the network included “crowding, upper arch”, “ANB,” and “curve of Spee”, reflecting the clinical factors most influencing these decisions. Finally, the study aimed to be an aid and guide for planning therapies, especially for younger orthodontists.

Recent studies have analyzed the ability of some machine learning (ML) models to suggest orthodontic extractive treatments in heterogeneous populations. Leavitt et al. used 55 cephalometric and demographic inputs from 366 patients and used Random Forest (RF), Logistic Regression (LR), and Support Vector Machine (SVM) algorithms to forecast specific extraction patterns [43]. The best class accuracy was achieved for the upper-and-lower first-premolar pattern (81.63% with Random Forest). For the other premolar extraction patterns, they did not have a good prediction. Overjet, molar crowding, and molar relationship were considered by this study to be the best extraction indicators.

The study by Del Real et al. analyzed the ability of an automated machine learning model (Auto-WEKA) to predict the need for extractions on a sample of 14 patients based on gender, model variables, and cephalometric values [44]. It has been noted that combining model and cephalometric data yielded the highest accuracy of 93.9% (versus 87.4% with model data alone and 72.7% with cephalometric data alone). The authors observed that the model variables contributed more to the treatment prediction than the cephalometric measurements but stated that the prediction models achieved the highest levels when both were used. Artificial intelligence tools should serve as additional resources to support extraction decisions, offering a simplified and predictive treatment model [42].

In the context of orthognathic surgery, AI demonstrates notable diagnostic precision. Large-scale facial analysis frameworks, such as the model introduced by Paul G. M. Knoops, automatically classify craniofacial morphology, assist in identifying candidates for specialist referral, and predict postoperative outcomes, thereby streamlining surgical planning [45].

Complementary research using AI-based statistical models has provided valuable insights into the prognosis of skeletal Class III malocclusion, showing how specific subtypes correlate with distinct treatment paths and risks of failure with orthodontics alone [46].

Together, these studies highlight AI’s capacity to objectively assess facial shape, optimize surgical planning, and support clinicians in identifying the most appropriate treatment modality.

Despite these advancements, AI has not yet consistently surpassed the accuracy and reliability of experienced clinicians. A systematic review published in 2025 emphasizes that human supervision remains essential to ensure that AI-generated recommendations align with clinical judgment and individual patient needs. Because many AI systems are trained using expert opinion as the reference standard, inherent variability persists. Consequently, AI should be regarded as a decision-support tool rather than a replacement for clinical expertise. Future research should prioritize improving model generalizability, particularly for diverse patient populations and complex clinical scenarios [6].

## 6. Use of AI for Clear Aligners

The use of artificial intelligence has proven particularly valuable in clear aligner therapy, as AI-driven systems and predictive models have demonstrated a strong capacity to streamline workflow (Figure 1), enhance the precision of digital treatment planning, and enable greater customization based on each patient’s unique dental characteristics [7].

By improving both the analytical and operational aspects of aligner design and sequencing, AI contributes to more efficient processes and potentially more predictable treatment outcomes.

Among the key applications of artificial intelligence in clear aligner therapy are tooth segmentation, digital model registration, digital setup, and remote monitoring [7].

Tooth segmentation is a fundamental step, allowing the accurate identification and separation of each tooth from intraoral scans or CBCT datasets. AI-based segmentation can automate this traditionally time-consuming manual process, achieving high accuracy while reducing the need for manual refinement. These tools are particularly effective in patients with mild crowding and no dental restorations, offering results comparable to manual techniques while improving speed and efficiency, ultimately enhancing clinical workflow and treatment planning [47,48,49].

Digital model registration represents another critical application, enabling the integration of multiple data types, such as intraoral scans and CBCT images, into a unified 3D framework.

Deep learning algorithms can detect anatomical landmarks and match corresponding surfaces across datasets, ensuring precise fusion of dental and skeletal structures: this automated integration minimizes manual adjustments, supports accurate virtual treatment planning, and facilitates the creation of well-fitting aligners while allowing safe and predictable tooth movements [50,51].

The digital setup further supports treatment planning by allowing clinicians to visualize the final occlusion, and while it is part of the manufacturing process, the setup provides an additional data point that informs decisions such as interproximal reduction (IPR) or the placement of composite resin attachments, helping refine and optimize the treatment plan [52].

**Figure 2:**
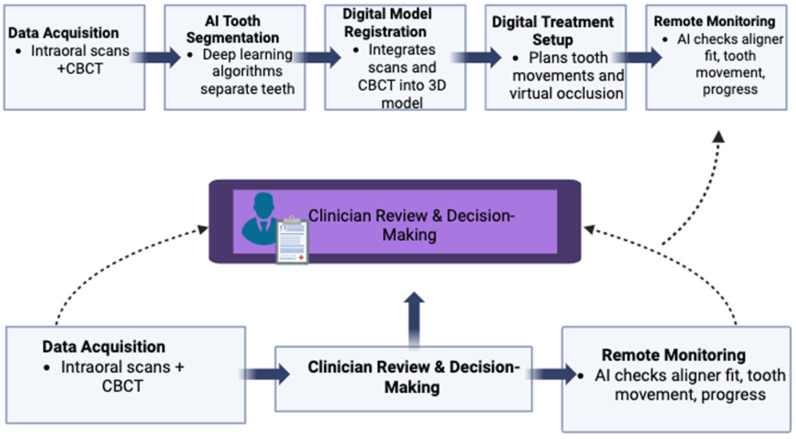
Schematic workflow of artificial intelligence in clear aligner therapy. AI automates key steps from imaging and tooth segmentation to treatment planning, aligner fabrication, and remote monitoring, while clinician supervision ensures accurate decision-making. Created with https://BioRender.com.

Artificial intelligence supports clinical decision-making through predictive analytics, enabling precise measurements, estimation of arch forms, prediction of tooth and root positions, and evaluation of patient-specific factors such as anxiety during clear aligner therapy, and these capabilities have been shown to improve treatment planning and support more predictable clinical outcomes, although some software systems still require refinement to increase the accuracy of tooth movement predictions [7] (Figure 2).

## 7. AI and Dental Scanner Devices: Treatment Simulation and Guided Acquisition of 2D and 3D Imaging

Artificial intelligence is reshaping dental impression taking by automating landmark detection, enhancing scan accuracy, and reducing human bias. Despite the advantages you certainly have, the precision of intraoral digital scanning can be influenced by several factors. The in vitro study by Răuță et al. [53] compared two of the most used intraoral scanners (Medit i700 and Trios 5) using an ISO 20896-1 test. According to the results, the Medit i700 stands out for its more consistent accuracy and decreased variability, validated by the lower values of standard deviation and variance. Despite this, the Trios 5 exhibits superior temporal performance, particularly in the “S” type scanning strategy, highlighting a greater dispersion of data, which could mean greater sensitivity to the operator’s technique. The results indicate that both intraoral scanners that were evaluated can give reliable results, with the Medit i700 less operator-dependent and achieving greater consistency. Consequently, the authors recommend using a standardized scanning protocol and selecting a scanner that matches the clinical workflow to ensure reliable full-arch digital impressions.

The in vitro study by Inal et al. [54] compared six contemporary intraoral scanners (IOSs) for mandibular implant-supported hybrid prostheses. Statistical analysis confirmed significant inter-scanner differences and rejected the null hypothesis that all IOSs perform equivalently: a better performance was achieved by the Primescan and Trios systems regarding trueness and precision respectively.

In order to identify the real reliability of the new scanners, studies were carried out in the literature aimed at comparing the difference in recording occlusal contacts between an intraoral scanner and a normal carbon paper: this is what was done by Didier et al. [55]. The study evaluated occlusal contact registration in 35 patients using an iTero Element intra-oral scanner and 8 µm carbon paper. Scanning produced 1548 contact regions (48% red, 52% orange) whereas carbon paper recorded 917 contacts. Overall agreement between the two methods was poor, with most occlusal contacts located in the posterior regions: integrating scanner data with carbon paper markings provides a more accurate occlusal assessment.

Collecting behavioral data and providing personalized feedback play a key role in improving treatment outcomes. Integrating AI into these processes can strengthen patient compliance and optimize overall orthodontic results, ensuring that digital therapy is not only efficient and precise but also responsive to individual patient needs [51].

Nowadays, there is the possibility, provided by some scanners and clear aligners, to predict what the final result of the treatment could be: some AI software can insert a simulated smile after treatment into the patient’s face photo, allowing the patient to immediately visualize what the final result could be (Figure 1).

The article by Adel et al. [56] compared the digital smile simulation performed pre-treatment by AI software *Invisalign SmileView*™ with the actual result obtained after a treatment of 18 ± 6 months, with upper and lower clear aligners. The system used photo uploading using an AI-enabled application, which then created a simulation of the patient’s final smile. For the five variables evaluated (philtrum height, commissure height, smile width, buccal corridor, and smile index), the actual values were very similar to those of the pre-treatment prediction. Among the qualitative variables, only the lip line variable was comparable between the simulation and the actual result. The *p* value was shown to be significant for intercanine width, thus showing that this value cannot be considered reliable for smile prediction.

The *Invisalign SmileView*™ AI tool was also analyzed in another study, which aimed to evaluate whether this artificial intelligence system can actually predict orthodontic outcomes and whether it creates anatomical changes in teeth, and whether it can align the facial and dental midline [57]. An initial social smile photo was taken of the 51 subjects included in the study, which was then compared to the new smile obtained after treatment. This SmileView system was able to simulate broader smiles, more easily treated with clear aligners. Moreover, it showed high predictability regarding the vertical movement of the incisors and adjusted the mesiodistal dimension of the upper incisors in its simulations. The software modified the mesiodistal proportion of the upper incisors, aiming for a “golden ratio” of 0.72, thus altering the real dental dimensions. However, it did not significantly alter gingival exposure, although a slight improvement was observed. It correctly identified deviations in dental midlines from the facial midline, but with a greater margin of error in proposed corrections for the lower arch.

One aspect that should be considered, however, is that smile arch prediction should not be limited to a facial aesthetic assessment based on the photos and digital scan, but should also include the cephalometric analysis values, to best predict the result that can be obtained. In fact, there is otherwise a risk of immediately showing the patient a result that, in reality, may not be possible due to anatomical limitations that can only be assessed through cephalometry and not just orthodontic objective examination.

AI-generated Digital Smile Design is believed by several studies in the literature to provide facial aesthetic results, but future studies may be useful to effectively investigate the cost-effectiveness of using AI in dentistry [58]. Although AI has helped improve workflow, the human intervention provided by an experienced doctor remains critical, and in the future, we should combine AI automation with clinician-suggested personalized intervention [59].

## 8. AI and Patient Communication in Orthodontics

The use of artificial intelligence in orthodontics has also changed the relationship between dentists and patients, particularly the communication between them (Figure 1). AI-based software allows the creation of individualized 3D models of the patient in which the teeth and jaw are visualized and on which the treatment is scheduled. In this regard, Chiang et al.’s article proposed a 3D communication model between patient and dentist, so that the dentist can modify the virtual model and show the patient the treatment they will be undergoing, including dental movements and the final change in the patient’s face [60]. The results of qualitative interviews with dentists and of patient questionnaires showed positive results: the system proved to be an effective tool for communication between dentists and patients, increasing satisfaction.

Studies have shown that the relationship with the patient is one of the most important factors for the patient’s satisfaction [61] and that good communication between patient and orthodontist affects the success of the treatment performed [62,63].

Moreover, tele-orthodontics, the technology that enables remote communication and monitoring, has proven to be a useful tool in achieving patient satisfaction [64].

In conclusion, the visualization for patients of the entire treatment process by AI-generated 3D models helps them set realistic expectations and also increases their motivation and adherence to treatment [65].

### 8.1. Dental Monitoring

Remote monitoring in clear aligner therapy is another area where AI demonstrates substantial clinical value, and by analyzing images captured on a patient’s smartphone, AI systems can assess whether a patient is ready to progress to the next aligner and detect deviations in tooth movement by identifying gaps between the aligner and teeth. Evidence shows that virtual monitoring can reduce the number of in-office visits, especially for younger patients, without prolonging treatment or increasing the number of aligners required. Patients report high satisfaction with these systems, perceiving them as easy to use, supportive, and reassuring, as they allow continuous virtual oversight [66,67].

Dental Monitoring (DM) is one of the most widely used commercial platforms for remote orthodontic supervision, based on convolutional neural networks (CNNs) that analyze intraoral photographs or videos captured by patients through a dedicated mobile application [68].

The system combines a patient-oriented app, a movement tracking algorithm, and a web-based dashboard, allowing clinicians to remotely review and manage treatment progress [69].

By enabling continuous monitoring, DM facilitates the early detection of clinical issues such as aligner misfit, appliance damage, inadequate oral hygiene, including plaque accumulation, gingival inflammation, and carious lesions, and poor patient compliance. Automated alerts sent to both patients and clinicians further reinforce adherence and encourage patients to follow proper treatment behaviors, such as active smartphone reminders that help maintain compliance and oral hygiene throughout orthodontic treatment [67,69].

Evidence shows that DM notifications significantly improve patient oral hygiene during treatment with fixed appliances: patients using DM scans demonstrate better plaque control and gingival health compared to controls, although long-term indices gradually increase, with a slight plateau observed around 4–6 months [70].

AI-powered systems enhance the ability to identify subtle deviations or emerging problems by comparing real-time tooth movements with predicted patterns, allowing timely interventions, such as adjustments to aligners or bracket positioning, to prevent delays or more extensive corrective procedures [71]. These features are particularly valuable for patients with clear aligners, those with braces, or individuals who live far from the clinic or have limited availability for frequent visits [71]; in fact DM contributes to a significant reduction in in-office appointments while maintaining effective clinical oversight, including during the retention phase [67].

The availability of remote orthodontic apps is rapidly expanding, with platforms such as the Apple Store offering over 300 applications for download, highlighting the growing interest and accessibility of digital monitoring tools in clinical practice [7]. Patients of different ages and abilities can generally perform scans successfully when standardized photographic techniques and appropriate tools are used [69,71]. Proper aligner use remains critical, as wearing aligners less than 22 h per day can lead to “non-tracking,” in which the appliance no longer fits the teeth correctly. TeleHealth systems like DM, combined with scan boxes, enable home-based scans after an initial clinic-guided setup, with immediate “GO” or “NO-GO” feedback to guide treatment progression [72]. Complementary apps such as StrojCHECK further enhance adherence by tracking behaviors, sending reminders, providing education, and motivating patients, with AI-driven updates improving clinical outcomes irrespective of patient age [72].

Future AI developments aim to detect non-compliance early, anticipate drops in discipline, optimize treatment plans based on individual behavioral and clinical profiles, and deliver personalized motivational interventions [72]. Moreover, novel solutions like the ARIA invisible aligner integrate flexible piezoelectric sensors with wireless electronics and machine learning algorithms to continuously monitor occlusal forces, bite patterns, and adverse oral habits [73]. By transmitting real-time data to smartphone interfaces, ARIA provides actionable feedback to patients, helping correct behaviors that could compromise treatment outcomes and enabling quantitative evaluation of retention and treatment effectiveness.

Overall, DM and AI-driven monitoring function as complementary tools within a hybrid care model, combining asynchronous remote supervision with periodic in-office examinations to ensure optimal treatment outcomes [67,69,71,72,74]. While most studies report no significant differences in overall treatment duration between patients monitored remotely and those receiving conventional follow-up, the number of required visits is consistently reduced with DM, offering clear advantages in terms of clinician efficiency, patient convenience, and reduced transportation-related costs [67]. Advanced AI applications, including robotic-assisted systems, have the potential to monitor multiple patients simultaneously, provide remote consultations, and adjust treatment plans, further enhancing personalized care [71].

### 8.2. AI and Chatbot

Since the advent of the internet, medical information has become increasingly popular among patients seeking clarification or alternatives to the treatments proposed by their doctors. This trend has also affected orthodontics and has followed the evolution of technology. Unable to control this phenomenon, researchers questioned the quality of information provided by internet search engines [72,73,74,75,76,77]. As of 30 November 2022, the emergence of ChatGPT (OpenAI Inc., San Francisco, CA, USA), a generative pretrained transformer (GPT), has changed the way users interact online when looking for information, including in the orthodontic field [78,79,80,81].

Artificial intelligence-based chatbots have emerged as versatile tools in orthodontics, with applications in both patient education and professional training. For patients, chatbots built on Large Language Models (LLMs) provide accurate, personalized guidance, reinforce oral hygiene practices, and support adherence to prescribed treatment behaviors, helping improve compliance when the information is validated [75]. In professional education, AI chatbots are explored as interactive learning aids for dental students and clinicians. Evaluations indicate that these systems can outperform students and general dentists in answering orthodontic questions, with certain versions, such as ChatGPT-4o, achieving accuracy levels close to those of experienced orthodontists [76]. However, consistency varies across models: ChatGPT-4 demonstrates the most reliable responses, whereas ChatGPT-4o, Claude 3.5 Sonnet, and Google Gemini 1.5 Pro show lower reproducibility, highlighting the potential risk of misinformation if used as the sole source of guidance [76]. Overall, while current chatbots are not yet capable of replacing traditional educational methods, they can complement conventional learning by promoting interactive engagement, critical thinking in diagnostic and treatment scenarios, and retention of knowledge. Continuous updates, expansion of question formats, and rigorous validation are necessary to maximize their educational value and reliability [75,76,77,78,79,80,81,82].

## 9. Emerging Perspectives in AI-Supported Orthodontics: From Biological Orthodontic Mechanism to Clinic

Recently, efforts have been made to apply AI to other aspects of orthodontic diagnostics and clinical practice, with the aim of speeding up and improving the quality of diagnostic tests and clinical performance.

AI, particularly deep learning and neural networks, excels in tasks such as automated cephalometric landmark detection, and in accurately classifying skeletal patterns. This capability streamlines diagnostic workflows and enhances reproducibility. Orthodontic treatment planning requires accurate assessment of patient’s skeletal maturity: in order to create a machine learning (ML) model to predict skeletal maturation, the study by Guo et al. [78] used the cervical vertebral morphology (six quantitative parameters) and dental maturation stage (DMS) of the mandibular second molar on routine lateral cephalograms and panoramic images, gender and age. Traditional CVM alone has shown limited diagnostic reliability, and hand–wrist films add radiation exposure, prompting the search for a less invasive yet accurate alternative. A total of 860 patients were included in the study: a machine learning workflow was built in Python 3.8.0 and six commonly used ML algorithms were compared. Among all of them CatBoost emerged as the best one. Adding DMS, gender, and chronological age to the baseline cervical-only model significantly boosted performance. The AI-driven model therefore provides a highly accurate method to estimate skeletal maturity and the prediction is better when DMS is combined with cervical morphology, age, and gender.

Artificial intelligence is moreover transforming the understanding of orthodontic tooth movement (OTM) and its underlying biological processes by enabling sophisticated data analysis and predictive modeling [79]. Machine learning algorithms and neural networks can detect patterns in genomic, radiographic and clinical records, enabling predictive models of how teeth will respond to applied forces. By integrating radiographs, 3D scans and patient-specific variables (e.g., genetics, bone density), AI provides personalized simulations of tooth movement, allowing the clinicians to choose the best therapy. This new way of thinking about OTM can change orthodontics [77].

One of the negative aspects that can occur following orthodontic treatment that involves the use of excessive force is external root resorption (external apical root resorption), which can most often occur in the upper maxillary incisors. In the study by Estrella et al. [80] artificial intelligence (AI) was applied to automate the three-dimensional segmentation of maxillary incisors from cone-beam CT scans, enabling rapid quantification of external apical root resorption. The study compared the conventional manual approach of segmentation with a fully automated one. The quantification of the root resorption obtained with the AI was similar to the manual method. Overall, AI facilitates the accurate detection of orthodontic-induced root resorption and demonstrates a positive impact in dentistry.

The application of artificial intelligence also extends to the assessment of the upper airway, enabling advanced analysis of airway morphology and volume in individual patients: there are interactive systems that clinicians can use to automatically generate three-dimensional segmentations of dentofacial and airway structures, quantitatively evaluate airway dimensions, and simulate anatomical relationships in a virtual environment, facilitating precise morphological assessment and support diagnosis [81].

**The study by Leonardi et al. [83] aimed to value the accuracy of a novel automatic approach based on a deep learning model based on CNN for the fully automatic segmentation of the sinonasal cavity and pharyngeal airway on CBCTs. The presented model proved to be comparable to that carried out by experts, but with greater speed.**

Another clinically significant application in airway analysis is the assessment of adenoid hypertrophy: deep learning algorithms can build an AI-driven airway segmentation and volumetric analysis, with advanced models applied to CBCT scans, that enable automated detection and evaluation of adenoid enlargement, supporting early OSA screening in pediatric patients. There are already models that have achieved sensitivity and specificity comparable to human experts, with a markedly reduced processing time, optimizing treatment planning [84].

One of the interesting additional factors regarding the use of artificial intelligence is its usefulness in fixed orthodontic therapy, particularly in the area of bracket placement and orthodontic archwire adjustment. It can be helpful in improving the efficiency and accuracy of automatic bracket positioning: the indirect bonding method (IDB) has especially shown high accuracy and precision in linear dimensions both with the traditional and digital methods, specifically useful for improving the accuracy of bracket angulation [82]. In the field of lingual orthodontics, which is particularly complex as it is characterized by uneasy visibility and access, indirect bonding trays have been analyzed in the literature: the study by Hoang et al. [85] analyzed the use of artificial intelligence in the design of 3D-printed indirect bonding trays, starting from digital intraoral scans of patients. Indirect bonding trays were automatically created using AI and patients were randomly divided into two groups: the first received lingual brackets bonded with grouped transfer trays and the second one with individual transfer trays. The results of the study showed clinically acceptable bracket transfer errors and grouped trays showed higher accuracy. In conclusion, the application of AI to improve orthodontic workflow is suggested, especially to improve orthodontic tools fabrication. The indirect bonding method and digital approaches streamline workflows by reducing laboratory steps and chair time and improving the overall efficiency for both the patient and the doctor [83].

The in vivo study by ElShebiny et al. [86] evaluated 840 brackets on 14 patients to compare direct bonding with virtual-indirect and AI bonding techniques approaches. Overall, both virtual methods (virtual-indirect and AI) showed statistically significant differences with direct bonding. However, the discrepancy between AI and clinician-guided virtual bonding was minimal (<0.25 mm), indicating no clinically relevant gap between the two digital workflows.

In conclusion there are several differences between direct and indirect bracket bonding: the first one places brackets manually on the patient’s teeth, a technique that is fast but highly operator-dependent and exposed to positional errors, whereas conventional indirect bonding—performed on a plaster model before the appointment—offers more precise placement and reduces chair-time, but still requires multiple laboratory steps and can be labor-intensive. The incorporation of artificial intelligence algorithms in the digital indirect bonding further enhances the workflow process: AI-driven virtual setups can automatically determine optimal bracket positions based on individualized treatment objectives, predict tooth movement, and provide real-time error checking, thereby increasing the accuracy of both indirect and, when used intraoperatively, direct bonding. By coupling AI-assisted planning with digital manufacturing, clinicians can achieve bracket placement that combines the efficiency of direct bonding with the precision of indirect techniques, ultimately delivering more predictable and personalized orthodontic outcomes.

Artificial intelligence could play an ever-increasing role in fixed therapy in the future, perhaps moving toward archwire adjustment by using sophisticated machine learning algorithms that translate the three-dimensional treatment plan into precise bending instructions. These models should analyze the desired torque, tip, and inter-arch relationships extracted from digital scans and predict the optimal curvature for each segment. AI-guided bracket placement, and automated wire fabrication could create a truly reliable and useful digital orthodontic workflow [81,84].

In conclusion, digital tools and artificial intelligence can provide valuable support to clinicians, both in diagnosis and in orthodontic treatment and follow-up.

## 10. Discussion

Despite these advances, critical reviews stress persistent challenges: most studies rely on homogeneous datasets, lack external validation, and only a single orthodontic AI device has received FDA clearance to date.

Addressing data heterogeneity, establishing standardized outcome sets, and conducting prospective multicenter trials are therefore essential to move AI from a promising adjunct to a reliable component of everyday orthodontic practice [3].

Therefore, the use of AI in dentistry has many advantages, such as increasing efficiency, reducing human risk, helping to make decisions, and improving its availability. Among its weaknesses, however, there are the total dependence on machines for its use, the large initial investment to use it, the lack of creativity, and finally, the ability to replace the human being, causing unemployment [87].

The uncontrolled and unrestrained use of AI can allow anyone to enter data into the algorithm to obtain a diagnosis, which could lead to the long-term risk of the machine replacing the doctor, who establishes a human relationship with the patient and offers them therapy. One solution to this scenario could be to propose a new medical paradigm in which AI helps the doctor in providing a diagnosis to the patient.

Recent evidence demonstrates that AI is already transforming multiple domains of orthodontics while complementing clinician expertise rather than replacing it [88]. In diagnostic imaging, deep learning algorithms, particularly convolutional neural networks (CNNs), have improved the speed and accuracy of cephalometric and CBCT analyses. Automated landmark detection has been shown to achieve comparable precision to experienced orthodontists, reducing inter- and intra-examiner variability and allowing clinicians to focus on higher-level decision-making. AI also enables automatic segmentation of hard and soft tissues, including teeth, mandible, maxilla, and airway structures, supporting precise diagnosis and comprehensive treatment planning [88,89].

In stereophotogrammetry, AI allows accurate localization of facial soft tissue landmarks, volumetric assessments, and integration with CBCT and intraoral scans to create complete virtual patient models. This technology has facilitated reproducible analyses of craniofacial symmetry, facial growth patterns, and aesthetic outcomes, which would otherwise be time-intensive and prone to human error [90,91].

In clear aligner therapy, AI is widely applied to tooth segmentation, digital model registration, treatment setup, and predictive movement simulations [92]. These automated processes streamline the workflow and reduce the need for manual refinement, while predictive algorithms enable personalized treatment plans. Studies report that AI-driven simulations, including smile visualization tools, can closely match post-treatment outcomes in terms of tooth positions and aesthetic parameters, improving patient understanding and engagement. Remote monitoring platforms such as Dental Monitoring allow patients to capture intraoral scans via smartphones, which AI algorithms evaluate for aligner fit, tooth movement, and hygiene compliance. Evidence shows that these systems maintain treatment efficacy while reducing in-office appointments, enhancing convenience, and supporting early detection of deviations. AI-driven alerts for misfit aligners, poor compliance, or oral hygiene issues further improve adherence and clinical outcomes [68,93].

AI has also shown promise in craniofacial growth prediction. Machine learning models can classify mandibular development, skeletal maturation, and post-pubertal growth trends with high accuracy. For example, convolutional neural networks applied to pre-treatment cephalograms have demonstrated 85–98% accuracy in predicting mandibular growth direction and skeletal relationships [32,94]. Predictive models using LASSO regression, Random Forest, and Support Vector Machines have shown consistent performance in longitudinal studies, particularly for skeletal landmarks [33]. Despite these successes, limitations remain in predicting soft tissue changes, dental inclinations, and the multidimensional complexity of growth, highlighting the need for larger, heterogeneous datasets and integration of additional clinical and familial variables.

Treatment planning and decision support are other areas where AI shows significant potential. Neural network models can replicate expert reasoning for extraction decisions, orthognathic surgery planning, and treatment sequencing. For instance, multilayer perceptron models have demonstrated high accuracy in predicting extraction necessity, extraction patterns, and anchorage strategies, supporting younger or less experienced clinicians in making evidence-based decisions. AI models can integrate multiple variables, including cephalometric measurements, crowding, incisor inclination, and facial profile, providing predictive outputs that aid but do not replace clinician judgment [95]. These systems are especially valuable in complex cases, heterogeneous populations, or when multiple treatment strategies are considered.

Despite these advancements, several challenges persist. AI performance is highly dependent on data quality and diversity. Misaligned radiographs, poor-quality CBCTs, and limited training datasets can reduce accuracy, particularly for linear measurements, posterior tooth movements, or soft tissue predictions. Generalizability is limited when algorithms are trained on homogeneous samples, emphasizing the need for multi-institutional datasets and standardization of imaging protocols. Additionally, most AI systems are validated against expert opinion, which introduces inherent variability. Ethical and legal considerations, including patient data privacy, algorithm transparency, and liability for AI-based decisions, remain critical concerns [96,97].

Patient engagement is another area where AI offers notable benefits through virtual treatment simulations and 3D models that improve communication, enabling patients to understand their treatment plan, set realistic expectations, and adhere more closely to prescribed regimens. Remote monitoring, smartphone applications, and AI-powered feedback foster continuous supervision and promote better oral hygiene, ultimately improving clinical outcomes and satisfaction [98].

In summary, AI should be regarded as a decision-support tool rather than a replacement for clinicians. Its strengths lie in efficiency, reproducibility, predictive capability, and enhanced patient engagement. However, limitations in dataset diversity, validation, and clinical oversight highlight that human expertise remains central. The optimal integration of AI in orthodontics is likely a hybrid model, combining automated algorithms with clinician supervision to achieve safe, personalized, and effective care. Future research should focus on validating AI across diverse populations, improving algorithm transparency, integrating multimodal data, and establishing standardized clinical protocols. By doing so, AI can maximize its potential to enhance orthodontic practice without compromising the essential human elements of clinical care.

## 11. Conclusions

Artificial intelligence is progressively transforming orthodontic practice by enhancing diagnostic accuracy, treatment planning, patient monitoring, and communication. As highlighted in this narrative review, AI-based systems demonstrate high potential in automating landmark identification, predicting craniofacial growth, optimizing clear aligner workflows, supporting extraction and surgical decision-making, and enabling efficient remote monitoring. These applications contribute to reduced operator variability, improved workflow efficiency, and increased patient engagement, particularly through visualization tools and tele-orthodontic platforms.

However, despite promising results, current evidence indicates that AI technologies have not yet reached a level of autonomy sufficient to replace clinical expertise. Limitations persist regarding dataset homogeneity, external validation, soft tissue prediction accuracy, and generalizability across diverse populations. Furthermore, ethical considerations, regulatory constraints, data privacy concerns, and the risk of over-reliance on automated outputs must be carefully addressed. The orthodontist’s role remains central in interpreting AI-generated data, integrating biological and psychosocial factors, and maintaining the human relationship that underpins patient-centered care.

In conclusion, artificial intelligence should be regarded as a powerful adjunctive tool rather than an autonomous decision-maker. Its optimal use lies in a synergistic model in which AI supports clinicians by improving efficiency and precision, while professional judgment ensures individualized, safe, and biologically sound treatment. These technologies have already demonstrated measurable improvements in diagnostic consistency, treatment planning accuracy, and workflow efficiency across a variety of orthodontic applications. Moreover, AI tools can enhance patient understanding and engagement by providing clear visualizations of treatment outcomes, supporting shared decision-making in clinical practice. Continued research, validation, and clinician training will be essential to fully integrate AI into routine orthodontic practice responsibly and effectively.

### Future Directions

Future advances in artificial intelligence in orthodontics will largely depend on the availability of large, diverse, and well-annotated datasets that reflect real-world clinical variability. Most current AI models are trained on homogeneous populations, often limited to specific ethnic groups, age ranges, or types of malocclusions. Expanding datasets to include different craniofacial patterns, growth stages, and treatment modalities will be essential to improve algorithm robustness and reduce bias. In parallel, the field is expected to move from retrospective single-center studies to prospective, multicenter clinical validation, allowing AI tools to be tested under routine clinical conditions and across different healthcare systems.

A key future direction is the development of multimodal AI systems capable of integrating heterogeneous data sources, such as cephalometric radiographs, CBCT scans, intraoral scans, 3D facial images, and clinical records. Rather than addressing isolated tasks (e.g., landmark detection or extraction decisions), next-generation AI platforms may function as comprehensive clinical decision-support systems, dynamically updating diagnostic assessments and treatment plans as new data become available. This approach could be particularly valuable in managing growth-related changes, treatment response variability, and long-term stability.

Another rapidly evolving area is explainable artificial intelligence, which aims to make algorithmic decision-making transparent and interpretable to clinicians. Improving explainability will be essential to foster trust, facilitate clinician oversight, and ensure that AI recommendations can be critically evaluated rather than passively accepted. This is particularly relevant in orthodontic treatment planning, where multiple valid therapeutic options often exist, and clinical judgment remains indispensable.

From a patient-centered perspective, future AI applications are expected to further enhance communication, motivation, and compliance through intelligent monitoring systems, adaptive feedback, and personalized behavioral support. Remote monitoring platforms may evolve to anticipate non-compliance, identify early deviations from planned tooth movements, and tailor motivational interventions to individual patient profiles. At the same time, conversational AI tools may become more integrated into clinical workflows, supporting patient education while remaining supervised by clinicians to prevent misinformation.

Ethical, legal, and regulatory considerations will play an increasingly central role in shaping AI adoption. Ensuring data privacy, preventing algorithmic bias, and defining professional accountability will be critical as AI systems become more autonomous and widely implemented. Regulatory frameworks are expected to evolve alongside technological innovation, emphasizing safety, transparency, and clinical validation. It is important to differentiate between commercial AI tools, which are typically regulated, clinically validated, and integrated into routine workflows, and research-grade models, which are often experimental and lack external validation. This distinction is essential when interpreting reported AI performance and assessing real-world applicability. In this context, obtaining regulatory clearance (e.g., FDA approval in the United States or CE in the European Union) will be essential before AI tools can be adopted for routine clinical use. Furthermore, medico-legal responsibility must remain clearly assigned, with clinicians retaining ultimate accountability for treatment decisions derived from AI-assisted recommendations. Clear guidelines on liability, documentation, and informed patient consent will be necessary to support safe and legally compliant AI integration [80].

The future of artificial intelligence in orthodontics is expected to involve a blended care model, where AI boosts efficiency, accuracy, and uniformity while maintaining the clinician’s essential role in diagnosis, decision-making, and patient care. Instead of replacing orthodontists, AI is anticipated to serve as a sophisticated cognitive assistant, aiding in the delivery of personalized, evidence-based, and ethically sound orthodontic practices.

## Figures and Tables

**Table 1:** Clinical readiness levels of artificial intelligence applications in orthodontics.

AI Application	Clinical Readiness	Description/Examples
Diagnostic imaging (lesion detection, landmark localization, jaw/airway/sinus segmentation)	Pilot	CBCT and 2D radiographs; algorithms show clinically acceptable accuracy for jaw, airway, and sinus segmentation [4].
Craniofacial growth assessment	Research	Predictive models trained on radiographs; currently experimental for clinical guidance datasets [5].
Extraction decision support	Research/Pilot	AI evaluates cephalometric data to suggest extractions; early-stage clinical testing [6].
Clear aligner treatment planning	Routine/Pilot	Widely used by manufacturers, AI programs predict tooth movements based on large datasets [7].
Digital impressions and CAD/CAM integration	Pilot	AI assists in scanning, modeling, and treatment design workflows [2].
Patient monitoring and communication	Pilot	AI supports tele-orthodontics, progress tracking, and visualization for patients [3].
Chatbots/online consultation tools	Research	Increasingly accessed by patients for information; currently advisory and not clinical [3,5].

## Data Availability

Not applicable.

## Abbreviations

AI	(artificial intelligence),
AIM	(artificial intelligence in medicine)
CAD	(computer-aided design)
CAM	(computer-aided manufacturing)
ASM	(active-shape-model)
CNN	(convolutional neural network)
DL	(deep learning)
ML	(machine learning)
SVR	(support vector regression)
AUC	(area under the curve)
IPR	(interproximal reduction)
DMS	(dental maturation stage)
IDM	(indirect bonding method)
OTM	(orthodontic tooth movement)

## References

[1] Patel V.L., Shortliffe E.H., Stefanelli M., Szolovits P., Berthold M.R., Bellazzi R., Abu-Hanna A. (2009). The coming of age of artificial intelligence in medicine. Artif. Intell. Med..

[2] Gandedkar N.H., Vaid N.R., Darendeliler M.A., Premjani P., Ferguson D.J. (2019). The last decade in orthodontics: A scoping review of the hits, misses and the near misses!. Semin. Orthod..

[3] Ahmed N., Abbasi M.S., Zuberi F., Qamar W., Halim M.S.B., Maqsood A., Alam M.K., Chohan H., Marya A., Itselis G. (2021). Artificial Intelligence Techniques: Analysis, Application, and Outcome in Dentistry—A Systematic Review. Biomed. Res. Int..

[4] Hung K., Yeung A.W.K., Tanaka R., Bornstein M.M. (2020). Current applications, opportunities, and limitations of AI for 3D imaging in dental research and practice. Int. J. Environ. Res. Public Health.

[5] El Naqa I., Keall M.R., Jin R., Jabbari K., Moore K., Miller J.M., Simpson I.J., Belderbos J.S.A., Sonke J.-J., Bradley J.D. (2018). Prospects and Challenges for Clinical Decision Support in the Era of Big Data. J. Oncol. Pract..

[6] Gracea R.S., Winderickx N., Vanheers M., Hendrickx J., Preda F., Shujaat S., Shaheen E., Politis C., Willems G., Jacobs R. (2025). Artificial intelligence for orthodontic diagnosis and treatment planning: A scoping review. J. Dent..

[7] Ruiz D.C., Mureșanu S., Du X., Elgarba B.M., Fontenele R.C., Jacobs R. (2025). Unveiling the role of artificial intelligence applied to clear aligner therapy: A scoping review. J. Dent..

[8] Arik S.Ö., Ibragimov B., Xing L. (2017). Fully automated quantitative cephalometry using convolutional neural networks. J. Med. Imaging.

[9] Kunz F., Stellzig-Eisenhauer A., Zeman F., Boldt J. (2020). Artificial intelligence in orthodontics: Evaluation of a fully automated cephalometric analysis using a customized convolutional neural network. J. Orofac. Orthop..

[10] Fukushima K. (1975). Cognitron: A self-organizing multilayered neural network. Biol. Cybern..

[11] LeCun Y., Bengio Y., Hinton G. (2015). Deep learning. Nature.

[12] Yasaka K., Akai H., Kunimatsu A., Kiryu S., Abe O. (2018). Deep learning with convolutional neural network in radiology. Jpn. J. Radiol..

[13] Yu H.J., Cho S.R., Kim M.J., Kim W.H., Kim J.W., Choi J. (2020). Automated Skeletal Classification with Lateral Cephalometry Based on Artificial Intelligence. J. Dent. Res..

[14] Jiang F., Guo Y., Yang C., Zhou Y., Lin Y., Cheng F., Quan S., Feng Q., Li J. (2023). Artificial intelligence system for automated landmark localization and analysis of cephalometry. Dentomaxillofac. Radiol..

[15] Cao L., He H., Hua F. (2022). Deep learning algorithms have high accuracy for automated landmark detection on 2d lateral cephalograms. J. Evid. Based Dent. Pract..

[16] Polizzi A., Lo Giudice A., Conforte C., Isola G., Leonardi R. (2025). Influence of head positioning errors on the accuracy of fully automated artificial intelligence-based cephalometric software. Angle Orthod..

[17] Hendrickx J., Gracea R.S., Vanheers M., Winderickx N., Preda F., Shujaat S., Shaheen E., Politis C., Willems G., Jacobs R. (2024). Can artificial intelligence-driven cephalometric analysis replace manual tracing? A systematic review and meta-analysis. Eur. J. Orthod..

[18] Polizzi A., Leonardi R. (2024). Automatic cephalometric landmark identification with artificial intelligence: An umbrella review of systematic reviews. J. Dent..

[19] Montúfar J., Romero M., Scougall-Vilchis R.J. (2018). Automatic 3-dimensional cephalometric landmarking based on active shape models in related projections. Am. J. Orthod. Dentofac. Orthop..

[20] Montúfar J., Romero M., Scougall-Vilchis R.J. (2018). Hybrid approach for automatic cephalometric landmark annotation on cone-beam computed tomography volumes. Am. J. Orthod. Dentofac. Orthop..

[21] Sarver D.M., Ackerman J.L. (2000). Orthodontics about face: The re-emergence of the esthetic paradigm. Am. J. Orthod. Dentofac. Orthop..

[22] Spampinato C., Pino C., Giordano D., Leonardi R. (2012). Automatic 3D segmentation of mandible for assessment of facial asymmetry. Proceedings of the 2012 IEEE Symposium on Medical Measurements and Applications Proceedings, Budapest, Hungary, 18–19 May 2012.

[23] Lo Giudice A., Ronsivalle V., Spampinato C., Leonardi R. (2021). Fully automatic segmentation of the mandible based on convolutional neural networks (CNNs). Orthod. Craniofac. Res..

[24] Tarce M., Zhou Y., Antonelli A., Becker K. (2024). The Application of Artificial Intelligence for Tooth Segmentation in CBCT Images: A Systematic Review. Appl. Sci..

[25] Baysal A., Sahan A.O., Ozturk M.A., Uysal T. (2016). Reproducibility and reliability of three-dimensional soft tissue landmark identification using three-dimensional stereophotogrammetry. Angle Orthod..

[26] Al-Baker B., Ayoub A., Ju X., Mossey P. (2024). Patch-based convolutional neural networks for automatic landmark detection of 3D facial images in clinical settings. Eur. J. Orthod..

[27] Berends B., Bielevelt F., Schreurs R., Vinayahalingam S., Maal T., de Jong G. (2024). Fully automated landmarking and facial segmentation on 3D photographs. Sci. Rep..

[28] Baksi S., Freezer S., Matsumoto T., Dreyer C. (2021). Accuracy of an automated method of 3D soft tissue landmark detection. Eur. J. Orthod..

[29] Al-Baker B., Alkalaly A., Ayoub A., Ju X., Mossey P. (2023). Accuracy and reliability of automated three-dimensional facial landmarking in medical and biological studies. A systematic review. Eur. J. Orthod..

[30] Björk A., Skieller V. (1983). Normal and abnormal growth of the mandible. A synthesis of longitudinal cephalometric implant studies over a period of 25 years. Eur. J. Orthod..

[31] Zhang J.N., Lu H.P., Hou J., Wang Q., Yu F.Y., Zhong C., Lu Y., Hou G. (2023). Deep learning-based prediction of mandibular growth trend in children with anterior crossbite using cephalometric radiographs. BMC Oral Health.

[32] Larkin A., Kim J.S., Kim N., Baek S.H., Yamada S., Park K., Lee Y.S. (2024). Accuracy of artificial intelligence-assisted growth prediction in skeletal Class I preadolescent patients using serial lateral cephalograms for a 2-year growth interval. Orthod. Craniofac. Res..

[33] Myers M., Brown M.D., Badirli S., Eckert G.J., Johnson D.H.M., Turkkahraman H. (2025). Long-Term Predictive Modelling of the Craniofacial Complex Using Machine Learning on 2D Cephalometric Radiographs. Int. Dent. J..

[34] Parrish M., O’Connell E., Eckert G., Hughes J., Badirli S., Turkkahraman H. (2023). Short- and Long-Term Prediction of the Post-Pubertal Mandibular Length and Y-Axis in Females Utilizing Machine Learning. Diagnostics.

[35] Kim E., Kuroda Y., Soeda Y., Koizumi S., Yamaguchi T. (2023). Validation of Machine Learning Models for Craniofacial Growth Prediction. Diagnostics.

[36] Zakhar G., Hazime S., Eckert G., Wong A., Badirli S., Turkkahraman H. (2023). Prediction of Pubertal Mandibular Growth in Males with Class II Malocclusion by Utilizing Machine Learning. Diagnostics.

[37] Wood T., Anigbo J.O., Eckert G., Stewart K.T., Dundar M.M., Turkkahraman H. (2023). Prediction of the Post-Pubertal Mandibular Length and Y Axis of Growth by Using Various Machine Learning Techniques: A Retrospective Longitudinal Study. Diagnostics.

[38] Kaźmierczak S., Juszka Z., Fudalej P., Mańdziuk J. (2021). Prediction of the facial growth direction with Machine Learning methods. arXiv.

[39] Kwon N., Kim J.-H., Suh H., Oh H., Lee S.-J. (2025). Factors influencing the predictive performance of artificial intelligence for craniofacial growth. Angle Orthod..

[40] Mohammad-Rahimi H., Nadimi M., Rohban M.H., Shamsoddin E., Lee V.Y., Motamedian S.R. (2021). Machine learning and orthodontics, current trends and the future opportunities: A scoping review. Am. J. Orthod. Dentofac. Orthop..

[41] Jung S.K., Kim T.W. (2016). New approach for the diagnosis of extractions with neural network machine learning. Am. J. Orthod. Dentofac. Orthop..

[42] Li P., Kong D., Tang T., Su D., Yang P., Wang H., Wang Z., Xiao L. (2019). Orthodontic Treatment Planning based on Artificial Neural Networks. Sci. Rep..

[43] Leavitt L., Volovic J., Steinhauer L., Mason T., Eckert G., Dean J.A., Dundar M.M., Turkkahraman H. (2023). Can we predict orthodontic extraction patterns by using machine learning?. Orthod. Craniofac. Res..

[44] Del Real A., Del Real O., Sardina S., Oyonarte R. (2022). Use of automated artificial intelligence to predict the need for orthodontic extractions. Korean J. Orthod..

[45] Knoops P.G.M., Papaioannou A., Borghi A., Breakey R.W.F., Wilson A.T., Jeelani O., Zafeiriou S., Steinbacher D., Padwa B.L., Dunaway D.J. (2019). A machine learning framework for automated diagnosis and computer-assisted planning in plastic and reconstructive surgery. Sci. Rep..

[46] Khosravi-Kamrani P., Qiao X., Zanardi G., Wiesen C.A., Slade G., Frazier-Bowers S.A. (2022). A machine learning approach to determine the prognosis of patients with Class III malocclusion. Am. J. Orthod. Dentofac. Orthop..

[47] Yacout Y.M., Eid F.Y., Tageldin M.A., Kassem H.E. (2024). Evaluation of the accuracy of automated tooth segmentation of intraoral scans using artificial intelligence-based software packages. Am. J. Orthod. Dentofac. Orthop..

[48] Al-Ubaydi A.S., Al-Groosh D. (2023). The Validity and Reliability of Automatic Tooth Segmentation Generated Using Artificial Intelligence. Sci. World J..

[49] Lee S.C., Hwang H.S., Lee K.C. (2022). Accuracy of deep learning-based integrated tooth models by merging intraoral scans and CBCT scans for 3D evaluation of root position during orthodontic treatment. Prog. Orthod..

[50] Cui Z., Fang Y., Mei L., Zhang B., Yu B., Liu J., Jiang C., Sun Y., Ma L., Huang J. (2022). A fully automatic AI system for tooth and alveolar bone segmentation from cone-beam CT images. Nat. Commun..

[51] Zheng Q., Wu Y., Chen J., Wang X., Zhou M., Li H., Zhang Y., Wang L., Sun X., Liu Y. (2025). Automatic multimodal registration of cone-beam computed tomography and intraoral scans: A systematic review and meta-analysis. Clin. Oral Investig..

[52] Hou D., Capote R., Bayirli B., Chan D.C.N., Huang G. (2020). The effect of digital diagnostic setups on orthodontic treatment planning. Am. J. Orthod. Dentofac. Orthop..

[53] Răuță S.-A., Vasilescu V.G., Ciocan L.T., Popa A., Țâncu A.-M.C., Froimovici F.O., Dimitriu B., Pițuru S.-M., Imre M. (2026). In Vitro Accuracy Analysis of Intraoral Scanning Strategies: A Comparison of Two Contemporary IOS Systems. Dent. J..

[54] İnal S., Uğur M. (2026). A comparative in vitro study of the accuracy and precision of different intraoral scanners for implant-supported hybrid prostheses impressions. BMC Oral Health.

[55] Didier V.F., de Miranda Ladewig V., de Freitas J.Q., Oltramari P.V.P., Fernandes T.M.F., de Almeida M.R., de Almeida Pedrin R.R., Henriques J.F.C., de Castro Ferreira Conti A.C. (2026). Concordance Between the Occlusal Contacts Record Obtained Using an Intraoral Scanner and Carbon Paper. Orthod. Craniofacial Res..

[56] Adel S.M., Bichu Y.M., Pandian S.M., Sabouni W., Shah C., Vaiid N. (2024). Clinical audit of an artificial intelligence (AI) empowered smile simulation system: A prospective clinical trial. Sci. Rep..

[57] Mourgues T., González-Olmo M.J., Huanca Ghislanzoni L., Peñacoba C., Romero-Maroto M. (2024). Artificial Intelligence in Aesthetic Dentistry: Is Treatment with Aligners Clinically Realistic?. J. Clin. Med..

[58] Saini R.S., Kaur K., Gurumurthy V., Binduhayyim R.I.H., Kaushik A., Kuruniyan M.S., Alarcón-Sánchez M.A., Heboyan A. (2025). Impact of artificial intelligence-based digital smile design on patient and clinician satisfaction and facial esthetic outcomes: A systematic review and meta-analysis. Digit. Health.

[59] Kaushik K., Sales A., Rodrigues S.J. (2025). Comparative analysis of facial aesthetics in AI generated versus conventionally crafted digital smile designs—A cross-sectional study. BDJ Open.

[60] Chiang Y.C., Wu F., Ko S.H. (2023). Effective Patient–Dentist Communication with a Simulation system for Orthodontics. Healthcare.

[61] Keles F., Bos A. (2013). Satisfaction with orthodontic treatment. Angle Orthod..

[62] Armfield J.M., Heaton L.J. (2013). Management of fear and anxiety in the dental clinic: A review. Aust. Dent. J..

[63] Panaite T., Romanec C.L., Adina A., Carina B., Savin C., Sîrghie A. (2025). Psychosocial Determinants of Patient Satisfaction in Orthodontic Treatment: A Pilot Cross-Sectional Survey in North-Eastern. Medicina.

[64] Almoammar S. (2024). The Role of Tele-Orthodontics in Enhancing Patient Compliance and Treatment Monitoring. J. Pharm. Bioallied Sci..

[65] Abutayyem H., Alsalam A., Iqbal R., Alkhabuli J., Mohamed S. (2019). Robotic Use in Orthodontics: Literature Review. Oral Health Dent. Sci..

[66] Ferlito T., Hsiou D., Hargett K., Herzog C., Bachour P., Katebi N., Tokede O., Larson B., Masoud M.I. (2023). Assessment of artificial intelligence-based remote monitoring of clear aligner therapy: A prospective study. Am. J. Orthod. Dentofac. Orthop..

[67] Martínez Gil-Ortega A., Cintora-López P., Pérez-Rodríguez L.M., Viñas M.J., Aragoneses J.M., Arrieta-Blanco P. (2025). Pilot case control evaluation of artificial intelligence assisted orthodontic monitoring and pediatric patient perception. Sci. Rep..

[68] Polizzi A., Serra S., Leonardi R., Isola G. (2025). Clinical Applications of Artificial Intelligence in Teleorthodontics: A Scoping Review. Medicina.

[69] Sharma A., Sharma T., Tomer G., Pattanaik S., Khattar B., Shrinivaasan N.R., Hegde A.M., Qari H.S. (2025). Assessment of AI-based monitoring method in assessing oral hygiene during orthodontic treatment. J. Orthod. Sci..

[70] Snider V., Homsi K., Kusnoto B., Atsawasuwan P., Viana G., Allareddy V., Gajendrareddy P., Elnagar M.H. (2023). Effectiveness of AI-driven remote monitoring technology in improving oral hygiene during orthodontic treatment. Orthod. Craniofac. Res..

[71] Olawade D.B., Leena N., Egbon E., Rai J., Mohammed A.P.E.K., Oladapo B.I., Boussios S. (2025). AI-Driven Advancements in Orthodontics for Precision and Patient Outcomes. Dent. J..

[72] Thurzo A., Kurilová V., Varga I. (2021). Artificial Intelligence in Orthodontic Smart Application for Treatment Coaching and Its Impact on Clinical Performance of Patients Monitored with AI-TeleHealth System. Healthcare.

[73] Feng H., Song W., Li R., Yang L., Chen X., Guo J. (2025). A Fully Integrated Orthodontic Aligner With Force Sensing Ability for Machine Learning-Assisted Diagnosis. Adv. Sci..

[74] Elnagar M.H., Nieberg-Baskin H., Becher M.K., Mellion Z.J., Borello B.R., Wilk B., Skafi R. (2025). Assessing an AI-driven remote monitoring system for reliable clinical photo acquisition and patient usability. J. World Fed. Orthod..

[75] Santonocito S., Cicciù M., Ronsivalle V. (2025). Evaluation of the impact of AI-based chatbot on orthodontic patient education: A preliminary randomised controlled trial. Clin. Oral Investig..

[76] Metin U., Goymen M. (2025). Information from digital and human sources: A comparison of chatbot and clinician responses to orthodontic questions. Am. J. Orthod. Dentofac. Orthop..

[77] Alhammadi M.S., Sawady M., Fayed M.M.S., Abdulrab S., Zawahry N.O.E., Fekry M.A., Alamer A.Y.H., Halboub E. (2026). Performance of four chatbots versus orthodontists in answering multiple-choice questions in orthodontics and dentofacial orthopedics. J. World Fed. Orthod..

[78] Guo M., Daraqel B., Ai D., Ma X., Dong Y., Hu Y., Zheng L. (2026). Prediction of skeletal maturity using machine learning based on multiple biological indicators. Am. J. Orthod. Dentofac. Orthop..

[79] Steinberg T., Jung B., Husari A., Bai S., Tomakidi P. (2025). Shaping Orthodontics of the Future: Concepts and Implications from a Cellular and Molecular Perspective. Int. J. Mol. Sci..

[80] Estrella N.-F., Alexandra D.-S., Yun C., Palma-Fernández J.C., Alejandro I.-L. (2025). AI-aided volumetric root resorption assessment following personalized forces in orthodontics: Preliminary results of a randomized clinical triaL. J. Evid. Based Dent. Pract..

[81] Shujaat S., Jazil O., Willems H., Van Gerven A., Shaheen E., Politis C., Willems G., Jacobs R. (2021). Automatic segmentation of the pharyngeal airway space with convolutional neural network. J. Dent..

[82] Bachour P.C., Klabunde R.T., Grünheid T. (2026). Usefulness of an artificial intelligence–assisted indirect bonding method for optimizing orthodontic bracket positioning. Angle Orthod..

[83] Leonardi R., Lo Giudice A., Farronato M., Ronsivalle V., Allegrini S., Musumeci G., Spampinato C. (2021). Fully automatic segmentation of sinonasal cavity and pharyngeal airway based on convolutional neural networks. Am. J. Orthod. Dentofac. Orthop..

[84] Dong W., Chen Y., Li A., Mei X., Yang Y. (2023). Automatic detection of adenoid hypertrophy on cone-beam computed tomography based on deep learning. Am. J. Orthod. Dentofac. Orthop..

[85] Hoang V., Vuong T.Q.T., Nguyen P.H., Pham N.H., Hoang K.L., Hoang T.T.T., Huynh N.C.P., Tran L.H.T., Tran H.T.H., Hoang N.K. (2025). AI-Assisted Design of 3D-Printed Lingual Indirect Bonding Trays: A Comparative Evaluation of Bracket Transfer Accuracy. J. Clin. Med..

[86] ElShebiny T., Cortés-Mercado J., Stefanovic N., Palomo J.M. (2025). Comparison Between Direct, Virtual Aided by Clinician and Artificial Intelligence Bonding Techniques in Orthodontics. Orthod. Craniofac. Res..

[87] Bonny T., Al Nassan W., Obaideen K., Al Mallahi M.N., Mohammad Y., El-Damanhoury H.M. (2023). Contemporary Role and Applications of Artificial Intelligence in Dentistry. F1000Research.

[88] Thakur S.M., Shenoy U., Hazare A., Karia H., Khorgade P., Nandeshwar N., Bhattacharya S. (2024). Transforming orthodontics with artificial intelligence: A comprehensive review. J. Adv. Dent. Pract. Res..

[89] Londono J., Ghasemi S., Shah A.H., Fahimipour A., Ghadimi N., Hashemi S., Khurshid Z., Dashti M. (2023). Evaluation of deep learning and convolutional neural network algorithms accuracy for detecting and predicting anatomical landmarks on 2D lateral cephalometric images: A systematic review and meta-analysis. Saudi Dent. J..

[90] Daoud S., Shhadeh A., Zoabi A., Redenski I., Srouji S. (2025). The Role of Digital Technologies in Personalized Craniomaxillofacial Surgical Procedures. Oral Maxillofac. Surg. Clin. N. Am..

[91] Munkwitz S.E., Shah H., Iglesias N.J., Quan H., Riveron S., Nayak V.V. (2025). A Review of Artificial Intelligence in Craniofacial Surgery: Clinical Applications Beyond 3D Printing. J. Craniofac. Surg..

[92] Liu C., Liu Y., Yi C., Xie T., Tian J., Deng P., Liu C., Shan Y., Dong H., Xu Y. (2025). Application of a 3D Fusion Model to Evaluate the Efficacy of Clear Aligner Therapy in Malocclusion Patients: Prospective Observational Study. J. Med. Internet Res..

[93] La Rosa S., Quinzi V., Palazzo G., Ronsivalle V., Lo Giudice A. (2024). The Implications of Artificial Intelligence in Pedodontics: A Scoping Review of Evidence-Based Literature. Healthcare.

[94] Lyros I., Vastardis H., Tsolakis I.A., Kotantoula G., Lykogeorgos T., Tsolakis A.I. (2025). Growth Prediction in Orthodontics: A Systematic Review of Past Methods up to Artificial Intelligence. Children.

[95] Peng J., Zhang Y., Zheng M., Wu Y., Deng G., Lyu J., Wang X., Sun J., Zhang L., Chen S. (2025). Predicting changes of incisor and facial profile following orthodontic treatment: A machine learning approach. Head Face Med..

[96] Lee J.-H., Kim Y.-T., Lee J.-B. (2024). Identification of dental implant systems from low-quality and distorted dental radiographs using AI trained on a large multi-center dataset. Sci. Rep..

[97] Kale P., Seth N., Verma S., Varshney D.K., Sharma S. (2024). Artificial intelligence in dental imaging: A new era of precision and predictive diagnosis. IP Int. J. Maxillofac. Imaging.

[98] Kaushik R., Rapaka R. (2024). A Patient-Centered Perspectives and Future Directions in AI-powered Teledentistry. Discoveries.

